# The value of using patient‐reported outcomes for health screening during long‐term follow‐up after paediatric stem cell transplantation for nonmalignant diseases

**DOI:** 10.1111/hex.13902

**Published:** 2023-12-15

**Authors:** Joëll E. Bense, Nicole Guilonard, Femke Zwaginga, Anne M. Stiggelbout, Marloes Louwerens, Hilda Mekelenkamp, Arjan C. Lankester, Arwen H. Pieterse, Anne P. J. de Pagter

**Affiliations:** ^1^ Department of Pediatrics, Willem‐Alexander Children's Hospital, Division of Stem Cell Transplantation Leiden University Medical Center Leiden The Netherlands; ^2^ Department of Biomedical Data Sciences, Medical Decision Making Leiden University Medical Center Leiden The Netherlands; ^3^ Erasmus School of Health Policy and Management Erasmus University Rotterdam Rotterdam The Netherlands; ^4^ Department of Internal Medicine Leiden University Medical Center Leiden The Netherlands

**Keywords:** health screening, patient‐centred care, patient‐reported experience, patient‐reported outcomes, stem cell transplantation, value‐based healthcare

## Abstract

**Introduction:**

The assessment of using patient‐reported outcomes (PROs) within comprehensive care follow‐up programmes, specifically focused on health screening, remains largely unexplored. PROs were implemented in our late effects and comprehensive care programme after paediatric hematopoietic stem cell transplantation (HSCT) for nonmalignant diseases. The programme focuses solely on screening of physical and mental health and on discussing PROs during the consultation.

**Methods:**

The primary method of this study was semistructured interviews to explore the perspective of both patients and healthcare providers' (HCP) on the use of PROs, which were thematically analyzed. Additionally, an explorative quantitative approach with patient‐reported experience measures (PREMS) was used, with a pretest–posttest design, to assess whether the use of PROs was accompanied by more patient‐centred care.

**Results:**

From the patient‐interviews (*N* = 15) four themes were extracted: use of PROs (1) help to discuss topics; (2) make the patients feel understood; (3) create a moment of self‐reflection; and (4) make consultations more efficient. Pre‐ and postimplementation analysis of PREMs (*N* = 40) did not show significant differences in terms of patient‐centeredness.

**Conclusion:**

Our results demonstrate the added value of integrating PROs for health screening purposes within the long‐term follow‐up programme after paediatric HSCT, as perceived by both patient and HCP. With the active use of PROs, patients are stimulated to consciously assess their health status.

**Patient Contribution:**

This study included patients as participants. Caregivers were approached if patients were below a certain age. Additionally, preliminary results were shared with all patients (including nonparticipants) during a patient conference day.

## 1 INTRODUCTION

Patient‐reported outcomes (PROs) are increasingly applied in hematopoietic stem cell transplantation (HSCT) for the purpose of collecting data for research or monitoring symptoms.^1^, ^2^ However, PROs can also be used to better understand the patients' needs, and to support shared decision‐making (SDM).^3^, ^4^ Integrating PROs into routine care offers the healthcare provider (HCP) the opportunity to identify essential topics and address problems early on, provide personalized support, make timely referrals, and consequently improve quality of care.^5^, ^6^ PROs have been incorporated into the late effects (LEEF) and comprehensive care follow‐up programme after paediatric allogeneic HSCT for nonmalignant diseases. The integration of PROs in this programme was part of the implementation of value‐based healthcare in this care path, aiming to enhance healthcare quality further.

**Figure 1:**
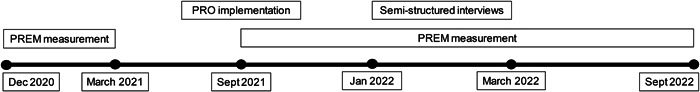
Overview measurements over time. Shown are the measurements within this study over time. The measurements involve PREMs, PRO measures, and semistructured interviews. PREM, patient‐reported experiences measures; PRO, patient‐reported outcome.

HSCT has proven to be an intensive, curative treatment option for various severe paediatric diseases, including nonmalignant disorders such as inborn errors of immunity, hemoglobinopathies and bone marrow failure syndromes.^7^, ^8^ Due to the HSCT procedure, consisting of chemotherapy and immunosuppressants, or due to the underlying disease, potential late effects can arise, such as gonadal dysfunction, renal insufficiency and cognitive problems, which consequently impair health‐related quality of life.^9^, ^10^, ^11^, ^12^, ^13^, ^14^ Proper screening for these late effects requires a dedicated long‐term follow‐up programme, which has been implemented at the Leiden University Medical Center (LUMC) in the Netherlands, providing comprehensive care from 2 years after HSCT onwards.^9^, ^10^ The programme includes annual monitoring of both physical and mental health (Supporting Information S1: Figure 1), and continues throughout adulthood due to the potential for late effects to occur even many years after paediatric HSCT.

Current research on the value of PROs in healthcare has predominantly focused on diseases where intervention efficacy, symptom control, or cure were the primary treatment objectives.^6^, ^15^ However, the value of PROs has not been investigated in care paths for screening programmes, where active healthcare utilization and overt disease symptoms may be absent. Therefore, the aim of this study was to explore patients', caregivers' and HCPs' experiences with the active use of PROs during consultations in the late effects and comprehensive care (LEEF) programme after paediatric HSCT for nonmalignant diseases. Furthermore, the study aimed to evaluate the impact of PRO use on patient‐centred care.

## 2 MATERIALS AND METHODS

### 2.1 PRO implementation

PROs were implemented in routine care in the LEEF programme in September 2021 (Figure 1). PRO domains from the International Consortium for Health Outcomes Measurement Standard Set ‘Overall Paediatric Health’ have been selected by consensus among both patients and the clinical team of the LEEF programme.^16^ Age‐appropriate and validated patient‐reported outcome measures (PROMs) were identified and selected based on their availability in Dutch (Supporting Information S1: Table 1). The validated Dutch‐Flemish PROMIS item banks used were Anxiety, Anger, Depressive Symptoms, Fatigue, Pain Interference, Pain Intensity, Sleep Disturbance, Mobility, Physical Function, Peer Relationships, Satisfaction with Social Roles and Activities and Cognitive Function.^17^, ^18^, ^19^, ^20^, ^21^, ^22^, ^23^, ^24^, ^25^, ^26^, ^27^, ^28^ Patients completed PROMs before their consultation using the digital KLIK PROM portal (www.hetklikt.nu).^29^ In addition, patients completed a symptom checklist (Supporting Information S1: Table 2). The HCP retrieved the PRO results in an electronic PROfile and discussed them with the patient during the consultation, for which the HCPs received training.^30^

### 2.2 Design

**Figure 2:**
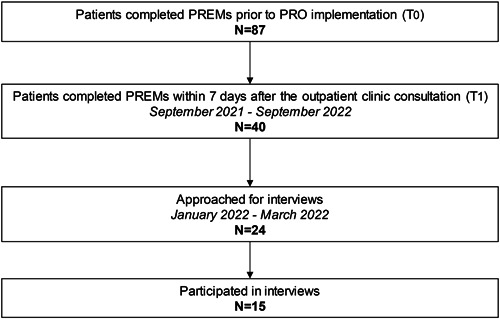
Flowchart showing inclusion of patients. Shown are the inclusion of patients in semistructured interviews and in the pretest–posttest design with PREMs. PREM, patient‐reported experiences measures; PRO, patient‐reported outcome.

The primary method of this study was semistructured interviews to explore the perspective of both patients and HCPs on the use of PROs. Additionally, in anticipation of changes related to patient‐centeredness, an explorative quantitative approach was used, with a pretest–posttest design, to assess whether the use of PROs was accompanied by more patient‐centred care (Figures 1 and 2).

### 2.3 Participants

Patients' inclusion criteria for both the interviews and the pretest–posttest study were: (1) allogeneic HSCT in childhood for a nonmalignant disease at the Willem‐Alexander Children's Hospital with a follow‐up of at least 2 years; (2) active follow‐up at the LUMC outpatient clinic (LEEF programme); (3) completion of PROMs before the consultation; (4) Dutch‐ or English‐speaking. Participants received complete study information and were recruited by telephone or in‐person. This study was approved by the medical ethics committee of Leiden—The Hague—Delft (N20.181). Written informed consent was obtained from all participants. For participants aged 15 years or younger, additional assent was obtained from (both) caregivers. All HCPs (*N* = 3) involved in the LEEF programme were included.

### 2.4 Measures

#### 2.4.1 Patient characteristics

Patient characteristics obtained from the medical files were age, gender and underlying disease (inborn errors of immunity, hemoglobinopathies and bone marrow failure disorders).

#### 2.4.2 Interviews with patients

Semistructured interviews were held from January to March 2022 to explore the patients' perspective on PRO use in all consecutive patients visiting the outpatient clinic. Participants were selected using convenience sampling. Interviews were held in‐person or by video conference, depending on the participant's preference. Two researchers (F. Z. and N. G.) who were not involved in the patient's care, conducted the interviews. For participants below the age of 12, one‐on‐one interviews were conducted with their parents, while participants ages 12 and above had the choice of being interviewed individually, with their parents, or together. The initial interview topic guide created by the researchers (J. B., H. M. and A. h. P.) was revised after the first three interviews, as it was found to focus excessively on the questionnaires themselves (PROMs) rather than the use of PROs during the consultation (Supporting Information S1: Table 3).

#### 2.4.3 Interviews with HCPs

To gain a comprehensive understanding of the use of PROs within the LEEF programme, HCPs were interviewed as well. Currently, three HCPs who use PROs work at the outpatient clinic. Two independent researchers (F. Z. and N. G.) took turns conducting the interviews with the HCPs. The topic guide created by researchers (J. B., F. Z. and N. G.) was adapted from the patients' topic guide (Supporting Information S1: Table 4).

#### 2.4.4 Patient‐reported experience measures (PREMs)

Two PREMs were used to assess if the use of PROs added value in terms of patient‐centred care. PREMs were selected by the research team based on expert opinion. The Person‐Centred Coordinated Care Experience Questionnaire (P3CEQ) consists of 11 items and is divided into two subscales: person‐centeredness (eight items), and care coordination (five items).^31^, ^32^, ^33^ The Revised Patient Perception of Patient‐Centeredness Questionnaire (PPPC‐R) consists of 18 items with three factors: (1) healthcare process (eight items); (2) context and relationship (eight items) and (3) roles (two items).^34^ Both PREMs were translated into Dutch language level B1 by the Dutch Centre of Expertise on Health Disparities (Pharos institute), and were approved by an independent test panel (*N* = 3). Participants completed the PREMs in the digital KLIK PROM portal on two separate occasions: *T*_0_) in before PRO implementation; *T*_1_) within 7 days after the outpatient clinic consultation (Figure 1). If participants were below 16 years of age, their caregiver completed the PREMs. Inclusion for the PREM analysis closed 1 year after PRO implementation, ensuring that all patients who participated in the *T*_0_ had the opportunity to participate in the *T*_1_ measurement.

### 2.5 Analysis

#### 2.5.1 Interviews

All interviews were recorded, transcribed verbatim and information was depersonalized. All interviews were conducted in Dutch. The participants' interviews were thematically analyzed using the Qualitive Analysis Guide of Leuven (Supporting Information S1: Table 5).^35^, ^36^, ^37^, ^38^ This guideline consists of a step‐by‐step method for analyzing qualitative data. Each researcher (J. B., F. Z. and N. G.) read and summarized the interviews, followed by the creation of conceptual interviews schemes. In these first steps, the relevant information from each interview is selected and clustered into different topics. After individual analysis, the researchers (J. B., F. Z. and N. G.) compared their findings, discussed their interpretation of the data, and reached consensus on a list of concepts (codes) linked to passages in each interview, enabling the identification of recurrent themes. Data collection and analysis took place simultaneously to enhance efficiency. Data collection continued until data saturation was reached, which was defined as no new upcoming themes in the analysis of the last three consecutive interviews. Finally, concepts were clustered into main themes and subthemes using the qualitative data analysis software ATLAS.ti (version 9).^39^ The HCPs' (*N* = 3) interviews were analyzed in the context of the themes identified from the patients' interviews. This approach was taken due to the small group size, which prevented data saturation from being achieved.

#### 2.5.2 PREMs

Statistical analysis of the PREM data was performed using SPSS version 25.^40^ Mean scores of P3CEQ and PPPC‐R at *T*_0_ vs. *T*_1_ were compared using paired sample *t*‐tests. A *p* < .05 was considered statistically significant. The PREM analysis was performed after the completion of interview analysis, and the PREM results were not available to the HCPs or interviewers. Participants who were interviewed completed both PREMs before the interview to avoid influencing the PREM scores.

## 3 RESULTS

### 3.1 Interviews with patients

In total, 15 of the 24 patients approached were interviewed after which data saturation was reached. Among the participants, eight out of 15 were male, ranging in age from 8 to 37 years (Table 1). The median interview duration was 21 min (range: 11–46 min). Supporting Information S1: Table 6 shows details from nonparticipants (*N* = 9). Upon coding and categorizing the data, four main themes emerged: (1) use of PROs help to discuss topics; (2) evaluating the PROs make the patients feel understood; (3) completing the PROs create a moment of self‐reflection; (4) use of PROs make the consultation more efficient. Additionally, participants were specifically asked about the usefulness of PROs and opportunities for improvement of use of PROs.

**Table 1:** Interviews: Patient characteristics (*N* = 15).

Characteristics	Median (range)
Gender, *N*	
Male	8
Female	7
Age at HSCT (years)	3 (1–15)
Age at interview (years)	17 (8–37)
Years since HSCT	11 (3–28)
Diagnosis, *N*	
Inborn errors of immunity	5
Hemoglobinopathies	5
Bone marrow failures	5
Second HSCT	2
Interview duration (min)	21 (11–46)
Interview setting, *N*	
Video conference	9
In person	6
Interview composition, *N*	
Participant	6
Parent of the participant	5
Participant and their parent	4

Abbreviation: HSCT, hematopoietic stem cell transplantation.

Most participants completed the questionnaires (PROMs) independently, while some younger participants required assistance from their caregivers. Due to COVID‐19 restrictions, participants over 18 years of age discussed the PROs with their doctor over the phone and were unable to view their results. Paediatric participants discussed their PROs during in‐person consultations at the LUMC and had the possibility to review their results together with the HCP as they discussed it. Illustrative quotations are given per theme (Tables 2, 3, 4, 5 and Supporting Information S1: Tables 7 and 8).

**Table 2:** Illustrative quotations from Theme 1 ‘Use of PROs help to discuss topics’.

Subtheme	Sex	Age	Quotation
Discussing the PROs to start the conversation	*♂*	11	*I think it provides [name HCP] the right tools to start a conversation, so that you don't have to start asking questions out of the blue*. [Caregiver about discussing PROs]
	*♀*	12	*… and then its looked upon to try to understand how the patient is going through life, if she is supported, if she is happy or not happy, if there are potential gaps, if she is deeply unhappy, you name it*. [Caregiver about discussing PROs]
	*♀*	17	*I thought it was better, because with the questionnaires you can really think clearly about everything beforehand and if you're at the appointment, well then you'll also forget half of it*.
	*♀*	30	*… it's nice, because then I can address it if I have something on my mind. And most of the time, as I said before, she acts upon this and takes action, so that's really nice*. [About discussing PROs]
Impact of discussing PROs	*♀*	13	*It's more like, all the HCPs know me better than I know them, so that makes it hard, because I only see them once a year*. [Caregiver about discussing emotionally charged subjects]
	*♂*	8	*The only thing that I'm thinking about is that, because your child is always sitting right next to you, I don't want to keep talking as if he is not there. So I consciously choose to not always discuss everything, except for when it's urgent, then I would dare to point that out*. [Caregiver about discussing certain topics]
	*♂*	37	*It's a difficult subject. And by confronting people that are not necessarily burdened by these feelings, or well, burdened is maybe too strong, but simply don't have these feelings, well, it's not making it easier*. [About PROs on depression]

Abbreviations: HCP, healthcare provider; PRO, patient‐reported outcome; ♀, female; ♂, male.

**Table 3:** Illustrative quotations from Theme 2 ‘Evaluating the PROs make the patient feel understood’.

Subtheme	Sex	Age	Quotation
Improvement of consultation preparation by the HCP	*♂*	37	*And she won't go through the questionnaire word for word, luckily, but I do notice that she asks substantive questions in such a way that I notice that she has read it, which I appreciate, because then I won't have done it for nothing and I can see that she is prepared*.
	*♀*	29	*When I filled it out, about how I felt in the last week and everything, so what you're supposed to do. I filled it in, and then [HCP] also responded to it. [HCP] did ask like: what's the reason you filled it out like this. So, I did find that nice*.
Patient feels supported	*♀*	16	*I just had the feeling that she like gets me and that she could relate with me, and I also appreciated the tips she gave me*. [About the HCP]
	*♀*	30	*I just feel really at ease when I come to you at the hospital. I mean, I really feel like a human being, you know, not just another number*.
	*♀*	20	*They help you understand what you mean exactly and they show a lot of commitment towards the questions you have*. [About the HCP]

Abbreviations: HCP, healthcare provider; PRO, patient‐reported outcome; ♀, female; ♂, male.

**Table 4:** Illustrative quotations from Theme 3 ‘Completing the PROMs create a moment of self‐reflection’.

Sex	Age	Quotation
*♂*	17	*He completed the questionnaires two days beforehand and well, then you talk about it, you talk about the whole process and about how his friends dealt with it and well, you get to have a moment in which you talk extensively about it*. [Caregiver]
*♀*	17	*I thought it was better, because with the help of the questionnaires you can really think clearly about everything*. [About filling in the PROMs]
*♀*	30	*Well, now we get the questionnaires that we have to fill in, so that's some sort of preparation*. [When asked about preparation before the consultation]

Abbreviations: PROM, patient‐reported outcome measure; ♀, female; ♂, male.

**Table 5:** Illustrative quotations from Theme 4 ‘Use of PROs make the consultation more efficient’.

Sex	Age	Quotation
*♂*	8	*I think it's beneficial that you don't have to discuss everything, then it's mostly the things you answered with yes or the things that are urgent, that are being highlighted and I think that is better, that saves time*. [Caregiver]
*♀*	21	*The other things, well the other answers I gave, were not different from the last time, so like, we didn't necessarily have to discuss these things*.

Abbreviations: PRO, patient‐reported outcome; ♀, female; ♂, male.

#### 3.1.1 Use of PROs help to discuss topics

##### Discussing the PROs to start the conversation

Almost all participants briefly reviewed their PROs with their HCP, had discussed them and had decided together which topics needed more clarification. The participants were satisfied with the way the PROs had been discussed. Discussing the PROs helped the participants to gain clarity about the questions they had and helped to facilitate the discussion. Participants felt that through discussing the PROs, the HCP can extract topics that are essential for the patient more easily and address questions that arose from the PROs. This was perceived as valuable. Furthermore, participants reported that completing the PROMs prepared them to talk about sensitive or personal topics, such as mental wellbeing, instead of feeling overwhelmed when the HCP initiates these topics without prior notice. Lastly, a few participants mentioned that discussing the PROs served as a reminder to address specific issues with their HCP.

##### Impact of discussing PROs

The majority of participants reported that when discussing the PROs they felt safe to discuss any topic they desired, including sensitive or personal topics. However, a few participants considered certain aspects of the PROs to be too personal and therefore did not want to discuss them during the consultation. Additionally, two parents preferred to discuss topics without their children present in the consultation room.

#### 3.1.2 Evaluating the PROs make the patients feel understood

##### Improvement of consultation preparation by the HCP

Many participants emphasized that PROs helped the HCP better prepare for the consultation. Additionally, they appreciated that the HCP already had insights into their emotional state. The participants felt that the HCP was able to focus more on their needs. One participant expressed the need to provide context on PRO‐related issues during the consultation to enhance the understanding of specific PROs.

##### Patient feels supported

Participants appreciated the time and attention of the HCP to evaluate the PROs. In addition, most participants experienced a sense of trust and support during their interactions with the HCP. Many participants appreciated that by evaluating the PROs, their well‐being was actively monitored and were overall satisfied with the consultation.

#### 3.1.3 Completing the PROs create a moment of self‐reflection

Completing the PROs prompted several participants to reflect on their current well‐being, their transplantation experience, and everything they have been through since then. It also helped them to reveal issues for which they needed support. Many participants perceived the request to complete the PROMs as a way to prepare for the consultation, as it invited them to reflect on essential aspects of their lives. Parents found that the PROs served as a conversation starter with their children on topics such as alcohol and drug use. However, a few participants perceived completing the PROs as mentally challenging and considered some questions as being too personal, such as the PROM regarding depressive symptoms.

#### 3.1.4 Use of PROs make the consultation more efficient

Some participants reported that using PROs made the consultation more efficient. The HCP was already aware of the most prominent issues, allowing irrelevant topics to be skipped or briefly touched upon, which was also preferred by half of the participants. A few participants noticed that their answers were directly transferred into their medical file, which meant the participants did not have to answer certain questions again.

#### 3.1.5 Additional results

Participants were specifically asked about the usefulness of PROs and opportunities for improvement of use of PROs.

##### Usefulness of PROs

While some participants did not personally perceive PROs as valuable because they did not have any problems to report, all participants emphasized the importance of PROs for those in need, healthcare improvements and research purposes. Some participants viewed the questionnaires within the context of active illness and treatment, and since they were already several years posttreatment, the PROs felt less relevant to them personally. However, participants could easily imagine that the PROs might be relevant to other patients. Most participants did not view completing the PROs as burdensome. Half of the participants regarded PROs as a valuable tool for monitoring the overall well‐being and health of all patients.

##### Opportunities for improvement of use of PROs

Nearly all participants considered the implementation of PROs to be an improvement of care. PRO content was clear and appropriate for the consultation. However, the number of questions in the PROMs was excessive and some participants preferred to skip topics they considered irrelevant. Certain questions were sometimes perceived as too personal and detailed for an online questionnaire. One participant also noted that certain PROMs emphasized negative aspects too much and lacked a positive approach. Some participants thought that the recall period of the PROMs was inappropriate and wished for an extended timeframe. Lastly, a few participants found the completion of PROs challenging due to the language level of the PROs and requested support from caregivers.

#### 3.1.6 Interviews with HCPs

Two of the three HCPs worked with paediatric patients and one with adult patients. The median interview duration was 44 min (range: 39–47). All HCPs perceived that the use of PROs improve the consultations, improve insight into patients' overall well‐being, and help to recognize and prepare topics needing attention during the consultation. The HCPs also noted that discussing the PROs led to more in‐depth conversations and made it easier to discuss personal subjects, such as sexuality, as patients had already reflected on them and were not caught off‐guard. However, the HCPs emphasized the need to verify patients' interpretation of the PROs and to conform or clarify any PRO‐related issues. The HCPs noticed that patients were better prepared for consultations and were more involved in their care. This resulted in improved equality and reciprocity in the HCP–patient interactions, and improved SDM after PRO implementation. However, one HCP perceived a sense of detachment due to patients answering personal questions online instead of in person. All HCPs reported that consultations became more efficient due to improved preparation, although the time requires for consultation preparation had increased both for HCPs and patients. Nevertheless, the HCPs experienced that with PROs, patients and HCPs were better prepared, facilitating SDM.

### 3.2 PREM

PRO implementation was evaluated by two PREMs (P3CEQ, PPPC‐R) regarding patient‐centeredness before (*T*_0_) and after PRO implementation (*T*_1_). *T*_1_ measurement ended 1 year after PRO implementation and included 40 patients. Twenty‐three patients were male. Age at *T*_1_ ranged from 6 to 42 years (Table 6). Mean scores at *T*_0_ and *T*_1_ from the P3CEQ and PPPC‐R were not significantly different (Table 7). There was a trend (*p* = .09) for more attention to the factor ‘context and relationship’ (PPPC‐R). Within this factor, per item analysis in this factor showed significant improvement in the perceived compassion from HCPs (mean [*T*_0_]: 1.3, SD: 0.6; mean [*T*_1_]: 1.1, SD: 0.5; 95% confidence interval [CI]: 0.01–0.4) and trust in HCPs (mean [*T*_0_]: 1.2, SD: 0.5; mean [*T*_1_]: 1.0, SD: 0.3; 95% CI: 0.04–0.4).

**Table 6:** PREM: Patient characteristics (*N* = 40).

Characteristics	Median (range)
Gender, *N*	
Male	23
Female	17
Age at HSCT (years)	6.3 (0.6–16.9)
Years since HSCT (years)^a^	9.5 (2.4–34.9)
Age at measurement (years)^a^	17.1 (5.8–41.6)
Diagnosis, *N*	
Inborn errors of immunity	12
Hemoglobinopathies	10
Bone marrow failures	18
Second HSCT, *N*	10

Abbreviations: HSCT, hematopoietic stem cell transplantation; PREM, patient‐reported experience measure.

^a^ Time was calculated from measurement *T*_1_.

**Table 7:** PREM: Mean PPPC‐R and P3CEQ scores before (*T*_0_) and after PRO implementation (*T*_1_).

	*T*_0_ mean (SD)	*T*_1_ Mean (SD)	*p*‐Value
P3CEQ^a^ (score range)			
Total (0–30)	19.0 (3.3)	19.6 (4.3)	.39
Person‐centred (0–24)	17.8 (2.9)	18.2 (3.3)	.47
Care coordination (0–15)	7.9 (2.6)	8.6 (2.5)	.18
PPPC‐R^b^ (score range: 1–4)			
Total	1.2 (0.4)	1.1 (0.4)	.37
Factor 1: Healthcare process	1.1 (0.5)	1.1 (0.5)	.95
Factor 2: Context and relationship	1.4 (0.5)	1.2 (0.4)	.09
Factor 3: Roles	1.2 (0.5)	1.1 (0.6)	.80

Abbreviations: P3CEQ, Person‐Centred Coordinated Care Experience Questionnaire; PPPC‐R, Revised Patient Perception of Patient‐Centeredness Questionnaire; PREM, patient‐reported experience measure; PRO, patient‐reported outcome.

^a^ A higher score indicates more of the component present.

^b^ A lower score indicates more of the component present.

## 4 DISCUSSION

This study aimed to explore the value of using PROs during consultations in the late effects and comprehensive care (LEEF) programme after paediatric HSCT for nonmalignant diseases. Four key themes emerged from the data. First, use of PROs helped to discuss topics and facilitate the conversation. Discussing the PROs guided an efficient consultation with a focus on the topics perceived as most relevant to the individual patient. Second, evaluating PROs made the patients feel understood and supported. The patient and HCP noticed mutual preparation before the consultation, resulting in more tailored follow‐up questions. Third, completing the PROs created a moment of self‐reflection for patients and parents. Fourth, use of PROs made the consultation more efficient due to better preparation. In addition to the four key themes, patients and caregivers had varying perceptions of the usefulness of PROs, both in positively and negatively.

When comparing our results to previous studies, several aspects must be considered, as PRO implementation and the use of PROs vary substantially across studies.^41^, ^42^, ^43^, ^44^ According to a review from Carfora et al.^44^ on the patient perspective regarding the use of PROs in clinical care, it is evident that various PROMs were utilized. The variations in PROMs used across studies can have implications for factors such as the length and number of questions in the PROMs, whether the PROMs were generic or disease‐specific, the extent to which the PROs were discussed during consultations, and whether visual aids were used to aid in the interpretation of PRO results. These variations could potentially influence patients' perceptions of the usefulness of PROs in their care. Nonetheless, there are many similarities between these studies and our results.

In line with previous research, use of PROs improved patient–physician communication, which could facilitate SDM.^44^, ^45^, ^46^ Although SDM was not explicitly addressed by patients or HCPs, there are elements of SDM that were highlighted in the interviews. Moreover, with the use of PROs, patients felt understood and supported by their HCP. HCPs reported a better understanding of their patient, enabling them to address personal topics more effectively. These factors could enhance the exploration of patient's values, thereby supporting SDM. Overall, research into the connection between SDM and the use of PROs in general has predominantly been conducted in a restricted range of care paths, rather than in care paths centred around health screening, such as the LEEF programme.^47^ Further research is needed to explore the association between the use of PROs and SDM.

Certain topics mentioned in the interviews also correspond to the quantitative analysis in this study. Although the evaluation using PREMs showed no significant difference in overall patient‐centeredness after PRO implementation, detailed analysis did reveal a trend for more attention to the factor ‘context and relationship’, specifically related to the perceived compassion from HCPs and trust in HCPs. This factor also evaluates to which extent patients are comfortable discussing their problems with their HCP. These topics have been especially positively highlighted in the interviews, providing further support to the observed trend in the PREMs. Unfortunately, due to the small sample size, possible significant differences could not be demonstrated.

PRO implementation impacted patients as well. Use of PROs was valuable for self‐reflection and made patients feel more in control of their care, which is in line with previous research.^44^, ^48^ However, the literature also suggests that self‐reflection could be potentially stressful for patients.^41^, ^44^ From our interview data, it remains uncertain whether self‐reflection had resulted in stress. In the days prior and after the consultation prompted some patients to reflect on their time of hospitalization and the initial months following their discharge. This often led them to engage in discussions about this with their family. Still, it is not evident if this type of reflection induced stress.

Regarding possible effects of the use of PROs in the consultations, HCPs reported increased efficiency due to use of PROs, which has been described in the literature.^44^, ^49^ However, in this study we did not measure the consultation duration nor the time devoted to certain topics. Further research is needed to assess the efficiency of the consultations when using PROs. There have been studies where PROs were utilized as a tool for evaluating active symptoms and determining the need for a health check at the hospital. However, providing context to PRO results was deemed essential by both patients and HCPs.^44^, ^50^ Additionally, HCPs expressed the preference for PROs to complement rather than replace regular consultations. The HCPs highlighted that PROs should be used as an additional resource in the overall care process, providing valuable information to enhance patient–physician communication.^44^, ^51^

This study has multiple strengths. First, this study is a multiple methods study. Most studies have either used quantitative measures or qualitative measures, such as focus groups. Second, the research team consisted of individuals from diverse professional backgrounds. This interdisciplinary collaboration brought different expertize and perspectives to the study, enhancing the identification of a wide range of themes and subthemes. However, there are also some limitations that should be acknowledged. First, this study did not conduct interviews before PRO implementation, precluding a comparison from pre‐ to postimplementation. Second, not all participants were able to review their PRO results since their consultation was conducted by phone. As a result, participants could not always determine whether the topics discussed were influenced by the PRO results. Third, in addition to PROMs patients completed a symptom checklist. Although the difference between PROMs and the symptom checklist was clear to HCPs, this may have been less clear to patients. The interviewers were aware of this issue and asked for clarification when necessary. Fourth, there was a lack of PREMs specifically aimed at evaluating PRO implementation. However, we expected differences regarding patient‐centeredness based on literature and expert opinions, and therefore chose the PREMs accordingly.^3^, ^44^ Fortunately, the themes derived from the interview data aligned with the PREM factors, providing additional support for the findings. Fifth, the sample size was too small to have sufficient power to assess changes regarding patient‐centeredness. However, our results suggest the late effects and comprehensive care programme might already perceived to be focused on patient‐centred care. This was evident when comparing the P3CEQ results to Dutch adults with a chronic condition, suggesting that the programme had already achieved a relatively high level of patient‐centeredness.^33^ It is possible that further improvement in patient‐centeredness might be challenging to detect, as the programme was already performing well in this aspect. Lastly, there might be a potential bias in the study due to exclusion of patients who did not complete PROs, possibly underestimating the perceived usefulness and difficulty of the PROMs. Topics addressed in the participants' interviews, such as language level, number of questions and personal inquiries, could be contributing factors. Reasons for not completing PROs should be further investigated.

## 5 CONCLUSION

Overall, our results indicate that the use of PROs for screening purposes in the late effects and comprehensive care programme after paediatric HSCT is valuable from both patient's and HCP's perspectives. It is important to note that completing PROs should not replace routine consultations, as patients and HCPs have expressed the importance of providing context to the PRO results. The use of PROs can lead to more efficient consultations by addressing the essential topics identified through PRO analysis. Moreover, with the active use of PROs there might be a shift towards a more mutual patient–HCP relationship, in which patients are stimulated to consciously assess their health status. Future research could focus on linking PRO results to psychosocial and clinical outcomes, enabling further optimization of PROs as a screening tool and provide valuable insights into the relationship between PROs and patients' overall well‐being.

## AUTHOR CONTRIBUTIONS

Joëll E. Bense approached the participants, collected hematopoietic stem cell transplantation data and performed statistical analysis. F. Zwaginga and N. Guilonard conducted the interviews. Joëll E. Bense, F. Zwaginga, N. Guilonard, Hilda Mekelenkamp and Arwen H. Pieterse prepared the topic guide and analyzed the data. Marloes Louwerens, Anne M. Stiggelbout, Arjan C. Lankester and Anne P. J. de Pagter provided expert opinion. Joëll E. Bense wrote the manuscript. All authors critically revised the manuscript, approved the final version to be published and agreed to accountability of the work.

## CONFLICT OF INTEREST STATEMENT

The authors declare no conflict of interest.

## ETHICS STATEMENT

This study was approved by the medical ethics committee of Leiden—The Hague—Delft (N20.181). Written informed consent was obtained from all participants. If participants were 15 years old or younger, additional assent was given by (both) caregivers.

## Supporting information

Supporting information.

## Data Availability

The data that support the findings of this study are available on request from the corresponding author. The data are not publicly available due to privacy or ethical restrictions.

## References

[1] Porter ME , Teisberg EO . Redefining Health Care: Creating Value‐Based Competition on Results. 6th ed. Harvard Business Review Press; 2006.

[2] Shah GL , Majhail N , Khera N , Giralt S . Value‐based care in hematopoietic cell transplantation and cellular therapy: challenges and opportunities. Curr Hematol Malig Rep. 2018;13(2):125‐134. 10.1007/s11899-018-0444-z 29484578 PMC6686881

[3] Porter I , Davey A , Gangannagaripalli J , et al. Integrating patient reported outcome measures (PROMs) into routine nurse‐led primary care for patients with multimorbidity: a feasibility and acceptability study. Health Qual Life Outcomes. 2021;19(1):133. 10.1186/s12955-021-01748-2 33902607 PMC8074460

[4] Weldring T , Smith SMS . Article commentary: patient‐reported outcomes (PROs) and patient‐reported outcome measures (PROMs). Health Serv Insights. 2013;6:HSI.S11093. 10.4137/hsi.S11093 PMC4089835 25114561

[5] Basch E , Barbera L , Kerrigan CL , Velikova G . Implementation of patient‐reported outcomes in routine medical care. Am Soc Clin Oncol Educ Book. 2018;38:122‐134. 10.1200/edbk_200383 30231381

[6] Dobrozsi S , Panepinto J . Patient‐reported outcomes in clinical practice. Hematology. 2015;2015:501‐506. 10.1182/asheducation-2015.1.501 26637765

[7] Passweg JR , Baldomero H , Chabannon C , et al. The EBMT activity survey on hematopoietic‐cell transplantation and cellular therapy 2018: CAR‐T's come into focus. Bone Marrow Transplant. 2020;55(8):1604‐1613. 10.1038/s41409-020-0826-4 32066864 PMC7391287

[8] Gooley TA , Chien JW , Pergam SA , et al. Reduced mortality after allogeneic hematopoietic‐cell transplantation. N Engl J Med. 2010;363(22):2091‐2101. 10.1056/NEJMoa1004383 21105791 PMC3017343

[9] Baker KS , Bresters D , Sande JE . The burden of cure: long‐term side effects following hematopoietic stem cell transplantation (HSCT) in children. Pediatr Clin North Am. 2010;57(1):323‐342. 10.1016/j.pcl.2009.11.008 20307723

[10] Shankar SM , Carter A , Sun CL , et al. Health care utilization by adult long‐term survivors of hematopoietic cell transplant: report from the bone marrow transplant survivor study. Cancer Epidemiol Biomarkers Prevent. 2007;16(4):834‐839. 10.1158/1055-9965.Epi-06-0714 17416780

[11] Shenoy S , Gaziev J , Angelucci E , et al. Late effects screening guidelines after hematopoietic cell transplantation (HCT) for hemoglobinopathy: consensus statement from the second pediatric blood and marrow transplant consortium international conference on late effects after pediatric HCT. Biol Blood Marrow Transplant. 2018;24(7):1313‐1321. 10.1016/j.bbmt.2018.04.002 29653206

[12] Heimall J , Buckley RH , Puck J , et al. Recommendations for screening and management of late effects in patients with severe combined immunodeficiency after allogenic hematopoietic cell transplantation: a consensus statement from the Second Pediatric Blood and Marrow Transplant Consortium International Conference on late effects after pediatric HCT. Biol Blood Marrow Transplant. 2017;23(8):1229‐1240. 10.1016/j.bbmt.2017.04.026 28479164 PMC6015789

[13] Lugthart G , Jordans CCE , de Pagter APJ , et al. Chronic kidney disease ten years after pediatric allogeneic hematopoietic stem cell transplantation. Kidney Int. 2021;100(4):906‐914. 10.1016/j.kint.2021.05.030 34102218

[14] de Kloet LC , Bense JE , van der Stoep MYEC , et al. Late endocrine effects after hematopoietic stem cell transplantation in children with nonmalignant diseases. Bone Marrow Transplant. 2022;57(10):1564‐1572. 10.1038/s41409-022-01755-x 35840745

[15] Dawson J , Doll H , Fitzpatrick R , Jenkinson C , Carr AJ . The routine use of patient reported outcome measures in healthcare settings. Brit Med J. 2010;340:c186. 10.1136/bmj.c186 20083546

[16] Algurén B , Ramirez JP , Salt M , et al. Development of an international standard set of patient‐centred outcome measures for overall paediatric health: a consensus process. Arch Dis Child. 2021;106(9):868‐876. 10.1136/archdischild-2020-320345 33310707 PMC8380885

[17] Quinn H , Thissen D , Liu Y , et al. Using item response theory to enrich and expand the PROMIS® pediatric self report banks. Health Qual Life Outcomes. 2014;12:160. 10.1186/s12955-014-0160-x 25344155 PMC4212129

[18] Amtmann D , Cook KF , Jensen MP , et al. Development of a PROMIS item bank to measure pain interference. Pain. 2010;150(1):173‐182. 10.1016/j.pain.2010.04.025 20554116 PMC2916053

[19] Lai JS , Cella D , Choi S , et al. How item banks and their application can influence measurement practice in rehabilitation medicine: a PROMIS fatigue item bank example. Arch Phys Med Rehabil. 2011;92(suppl 10):S20‐S27. 10.1016/j.apmr.2010.08.033 21958919 PMC3696589

[20] Irwin DE , Gross HE , Stucky BD , et al. Development of six PROMIS pediatrics proxy‐report item banks. Health Qual Life Outcomes. 2012;10:22. 10.1186/1477-7525-10-22 22357192 PMC3312870

[21] Buysse DJ , Yu L , Moul DE , et al. Development and validation of patient‐reported outcome measures for sleep disturbance and sleep‐related impairments. Sleep. 2010;33(6):781‐792. 10.1093/sleep/33.6.781 20550019 PMC2880437

[22] Forrest CB , Meltzer LJ , Marcus CL , et al. Development and validation of the PROMIS pediatric sleep disturbance and sleep‐related impairment item banks. Sleep. 2018;41(6):zsy054. 10.1093/sleep/zsy054 29546286

[23] Pilkonis PA , Choi SW , Reise SP , Stover AM , Riley WT , Cella D . Item banks for measuring emotional distress from the Patient‐Reported Outcomes Measurement Information System (PROMIS®): depression, anxiety, and anger. Assessment. 2011;18(3):263‐283. 10.1177/1073191111411667 21697139 PMC3153635

[24] Irwin DE , Stucky BD , Langer MM , et al. PROMIS Pediatric Anger Scale: an item response theory analysis. Qual Life Res. 2012;21(4):697‐706. 10.1007/s11136-011-9969-5 21785833 PMC3245753

[25] Lai JS , Zelko F , Krull KR , et al. Parent‐reported cognition of children with cancer and its potential clinical usefulness. Qual Life Res. 2014;23(4):1049‐1058. 10.1007/s11136-013-0548-9 24197478 PMC3967001

[26] Hahn EA , DeWalt DA , Bode RK , et al. New English and Spanish social health measures will facilitate evaluating health determinants. Health Psychol. 2014;33(5):490‐499. 10.1037/hea0000055 24447188 PMC4159098

[27] Dewalt DA , Thissen D , Stucky BD , et al. PROMIS Pediatric Peer Relationships Scale: development of a peer relationships item bank as part of social health measurement. Health Psychol. 2013;32(10):1093‐1103. 10.1037/a0032670 23772887 PMC3865609

[28] Reeve BB , McFatrich M , Mack JW , et al. Expanding construct validity of established and new PROMIS pediatric measures for children and adolescents receiving cancer treatment. Pediatr Blood Cancer. 2020;67(4):e28160. 10.1002/pbc.28160 31904157 PMC7147933

[29] Haverman L , van Oers HA , Limperg PF , et al. Implementation of electronic patient reported outcomes in pediatric daily clinical practice: the KLIK experience. Clin Pract Pediatr Psychol. 2014;2(1):50‐67. 10.1037/cpp0000043

[30] Haverman L , van Oers HA , van Muilekom MM , Grootenhuis MA . Options for the interpretation of and recommendations for acting on different PROMs in daily clinical practice using KLIK. Med Care. 2019;57(suppl 5):S52‐S58. 10.1097/mlr.0000000000001061 30985597

[31] Sugavanam T , Fosh B , Close J , Byng R , Horrell J , Lloyd H . Codesigning a measure of person‐centred coordinated care to capture the experience of the patient: the development of the P3CEQ. J Patient Exp. 2018;5(3):201‐211. 10.1177/2374373517748642 30214927 PMC6134538

[32] Lloyd H , Fosh B , Whalley B , Byng R , Close J . Validation of the person‐centred coordinated care experience questionnaire (P3CEQ). Int J Qual Health Care. 2019;31(7):506‐512. 10.1093/intqhc/mzy212 30508089 PMC6839368

[33] Rijken M , Close J , Menting J , et al. Assessing the experience of person‐centred coordinated care of people with chronic conditions in the Netherlands: validation of the Dutch P3CEQ. Health Expect. 2022;25(3):1069‐1080. 10.1111/hex.13454 35318778 PMC9122454

[34] Ryan BL , Brown JB , Tremblay PF , Stewart M . Measuring patients' perceptions of health care encounters: examining the factor structure of the revised patient perception of Patient‐Centeredness (PPPC‐R) Questionnaire. J Patient Cent Res Rev. 2019;6(3):192‐202. 10.17294/2330-0698.1696 31414031 PMC6675140

[35] Dierckx de Casterlé B , Gastmans C , Bryon E , Denier Y . QUAGOL: a guide for qualitative data analysis. Int J Nurs Stud. 2012;49(3):360‐371. 10.1016/j.ijnurstu.2011.09.012 21996649

[36] Braun V , Clarke V . Using thematic analysis in psychology. Qual Res Psychol. 2006;3(2):77‐101. 10.1191/1478088706qp063oa

[37] Baarda B , Bakker E , Fischer T , et al. Basisboek Kwalitatief onderzoek: Handleiding Voor Het Opzetten En Uitvoeren Van Kwalitatief Onderzoek. Vol 3. Noordhoff Uitgevers; 2013.

[38] Bailey DM , Jackson JM . Qualitative data analysis: challenges and dilemmas related to theory and method. Am J Occup Ther. 2003;57(1):57‐65. 10.5014/ajot.57.1.57 12549891

[39] ATLAS.ti . ATLAS.ti Scientific Software Development GmbH. Version 8; 2023.

[40] IBM Corp . IBM SPSS Statistics for Windows. Version 25; 2017.

[41] Graupner C , Kimman ML , Mul S , et al. Patient outcomes, patient experiences and process indicators associated with the routine use of patient‐reported outcome measures (PROMs) in cancer care: a systematic review. Supp Care Cancer. 2021;29(2):573‐593. 10.1007/s00520-020-05695-4 PMC7767901 32875373

[42] Recinos PF , Dunphy CJ , Thompson N , Schuschu J , Urchek 3rd JL , Katzan IL . Patient satisfaction with collection of patient‐reported outcome measures in routine care. Adv Ther. 2017;34(2):452‐465. 10.1007/s12325-016-0463-x 28000165

[43] Kargo AS , Jensen PT , Lindemann K , et al. The PROMova study comparing active and passive use of patient‐reported outcome measures in ovarian cancer follow‐up: effect on patient‐perceived involvement, satisfaction with care, and usefulness. Acta Oncol. 2021;60(4):434‐443. 10.1080/0284186x.2021.1891281 33651647

[44] Carfora L , Foley CM , Hagi‐Diakou P , et al. Patients' experiences and perspectives of patient‐reported outcome measures in clinical care: a systematic review and qualitative meta‐synthesis. PLoS ONE. 2022;17(4):e0267030. 10.1371/journal.pone.0267030 35446885 PMC9022863

[45] Detmar SB , Muller MJ , Schornagel JH , Wever LDV , Aaronson NK . Health‐related quality‐of‐life assessments and patient‐physician communication: a randomized controlled trial. JAMA. 2002;288(23):3027‐3034. 10.1001/jama.288.23.3027 12479768

[46] Mondesir FL , Zickmund SL , Yang S , et al. Patient perspectives on the completion and use of patient‐reported outcome surveys in routine clinical care for heart failure. Circ Cardiovasc Qual Outcomes. 2020;13(9):e007027. 10.1161/circoutcomes.120.007027 32862696 PMC7494517

[47] Damman OC , Jani A , de Jong BA , et al. The use of PROMs and shared decision‐making in medical encounters with patients: an opportunity to deliver value‐based health care to patients. J Eval Clin Pract. 2020;26(2):524‐540. 10.1111/jep.13321 31840346 PMC7155090

[48] Neff C , Wang MC , Martel H . Using the PDQ‐39 in routine care for Parkinson's disease. Parkinsonism Rel Disord. 2018;53:105‐107. 10.1016/j.parkreldis.2018.05.019 29853294

[49] Nic Giolla Easpaig B , Tran Y , Bierbaum M , et al. What are the attitudes of health professionals regarding patient reported outcome measures (PROMs) in oncology practice? A mixed‐method synthesis of the qualitative evidence. BMC Health Serv Res. 2020;20(1):102. 10.1186/s12913-020-4939-7 32041593 PMC7011235

[50] Boyce MB , Browne JP , Greenhalgh J . Surgeon's experiences of receiving peer benchmarked feedback using patient‐reported outcome measures: a qualitative study. Implement Sci. 2014;9:84. 10.1186/1748-5908-9-84 24972784 PMC4227108

[51] Riis CL , Jensen PT , Bechmann T , Möller S , Coulter A , Steffensen KD . Satisfaction with care and adherence to treatment when using patient reported outcomes to individualize follow‐up care for women with early breast cancer—a pilot randomized controlled trial. Acta Oncol. 2020;59(4):444‐452. 10.1080/0284186x.2020.1717604 32000559

