# Supplementary material for: The value of using patient‐reported outcomes for health screening during long‐term follow‐up after paediatric stem cell transplantation for nonmalignant diseases
Source: Health Expect. 2023 Dec 15;27(1):e13902. doi: 10.1111/hex.13902 (PMC10768862; doi:10.1111/hex.13902)
Supplement: Supplementary file 1 — Supporting information. [file HEX-27-e13902-s001.docx]

**The value of using patient-reported outcomes for health screening during long-term follow-up after pediatric stem cell transplantation for nonmalignant diseases - *Supplementary Information***


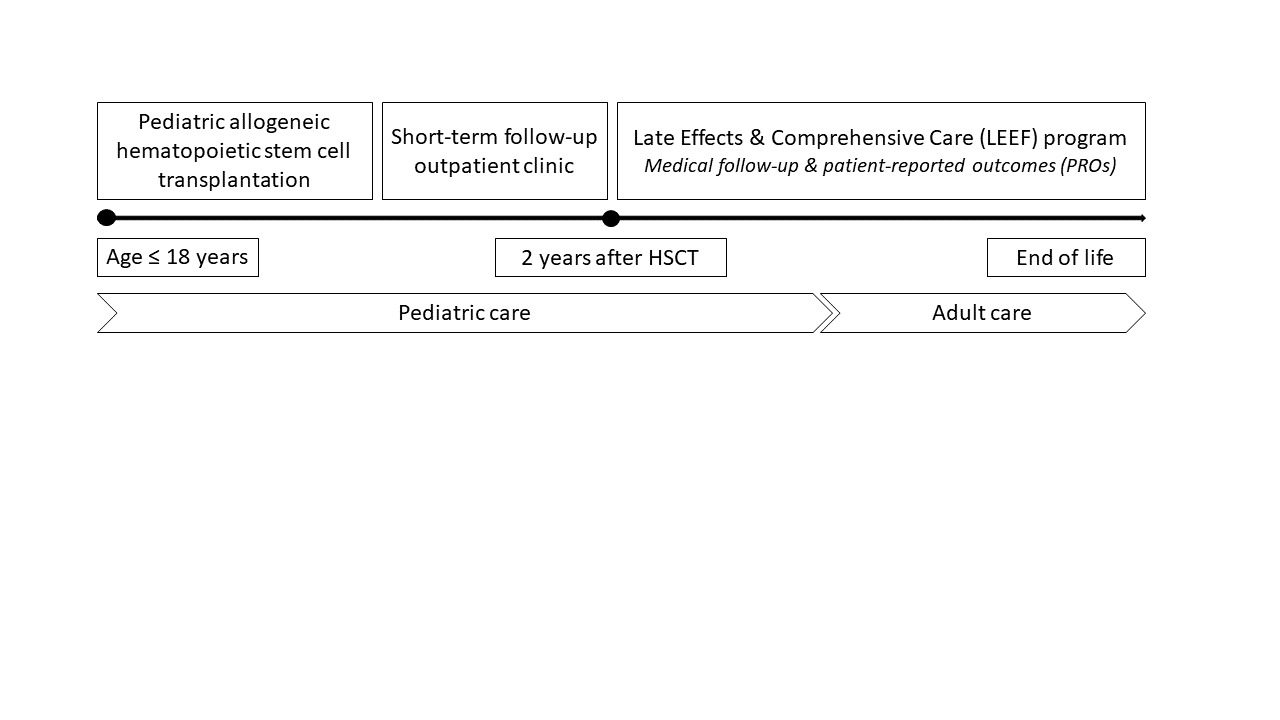


Supplementary Figure 1. Care path pediatric allogenic HSCT

Supplementary Table 1. Overview of PROMs per age category

| **PROM** | **2-4 years** | **5-7 years** | **8-12 years** | **13-17 years** | **18-30 years** | **≥31 years** |
| --- | --- | --- | --- | --- | --- | --- |
| *LEEF symptom checklist** | x | x | x | x | x | x |
| *PROMIS Anxiety* |  |  | x | x | x | x |
| *PROMIS Depressive Symptoms* |  | x | x | x | x | x |
| *PROMIS Fatigue* |  | x | x | x | x | x |
| *PROMIS Mobility* |  |  | x | x |  |  |
| *PROMIS Physical Function* |  |  |  |  | x | x |
| *PROMIS Pain Interference* |  |  | x | x | x | x |
| *PROMIS Pain Intensity* |  |  | x | x | x | x |
| *PROMIS Peer Relationships* |  | x | x | x |  |  |
| *PROMIS Satisfaction with Social Roles and Activities* |  |  |  |  | x | x |
| *PROMIS Sleep Disturbance* |  | x | x | x | x | x |
| *PROMIS Anger* |  |  | x | x |  |  |
| *PROMIS short form Cognitive function* |  | x | x | x |  |  |

** The LEEF symptom checklist was created by health care providers of the LEEF clinic.
PROMIS proxy measures (5-7 years) were CAT V2.0 – Depressive Symptoms, CAT V2.0 – Fatigue, CAT V2.0 – Peer Relationships, CAT V1.0 – Sleep Disturbance, Short Form V1.0 – Cognitive Function 7a, and Short Form V1.0 – Pain Intensity.
PROMIS pediatric measure (8-18 years) were CAT V2.0 – Anxiety, CAT V2.0 – Pain interference, CAT V2.0 – Depressive Symptoms, CAT V2.0 – Fatigue, CAT V2.0 – Peer Relationships, CAT V1.0 – Sleep Disturbance, CAT V2.0 – Mobility, Short Form V1.0 – Anger, Short Form V1.0 – Cognitive Function 7a, and Short Form V1.0 – Pain Intensity.
PROMIS adult measures (≥19 years) were CAT V1.0 – Anxiety, CAT V1.1 – Pain interference, CAT V1.0 – Depression, CAT V1.0 – Fatigue, CAT V2.0 – Satisfaction with Participation in Social Roles, CAT V1.0 – Sleep Disturbance, CAT V2.0 – Physical Functioning, and Short Form V1.0 – Pain Intensity*

Supplementary Table 2. LEEF symptom checklist

| **General** |  | |
| --- | --- | --- |
| What is the most important thing for you to discuss with your doctor? | *open response* | |
| **General** | Yes | No |
| Have you visited another doctor? If so, why? |  |  |
| Have you needed new medications in the past year? If so, which ones? |  |  |
| **General Health** |  |  |
| Are you in good physical shape compared to people your age? |  |  |
| Do you engage in sports? If yes, which ones? |  |  |
| **Head/Neck** |  |  |
| Have you had regular problems with your nose, mouth, throat, or lips since your last visit? |  |  |
| Do you go to the dentist? |  |  |
| Does your dentist know that you have had a stem cell transplant? |  |  |
| **Skin and Hair** |  |  |
| Have you had regular problems with your hair since your last visit? |  |  |
| Have you had regular problems with your skin since your last visit? |  |  |
| Have you had regular problems with your nails since your last visit? |  |  |
| **Heart, Lungs, and Kidneys** |  |  |
| *Have you been regularly experiencing the following issues since your last visit:* |  |  |
| Fainting |  |  |
| Chest pain or pressure |  |  |
| Heart palpitations |  |  |
| Swollen ankles (edema) |  |  |
| Difficulty breathing |  |  |
| Coughing |  |  |
| Urination |  |  |
| **Stomach and Intestines** |  |  |
| *Since your last visit, have you been regularly experiencing problems with:* |  |  |
| Swallowing |  |  |
| Heartburn/acid reflux or nausea |  |  |
| Defecation |  |  |
| Abdominal pain |  |  |
| **Movement** |  |  |
| *Have you been regularly experiencing the following issues since your last visit:* |  |  |
| Painful joints |  |  |
| Stiff or swollen joints |  |  |
| **Nerves** |  |  |
| *Have you been regularly experiencing problems or discomfort since your last visit:* |  |  |
| Headaches |  |  |
| Ears |  |  |
| Eyes |  |  |
| **Hormones** |  |  |
| Have you gained or lost a significant amount of weight since your last visit? |  |  |
| Have you felt unusually hot or cold since your last visit? |  |  |
| Have you been unusually thirsty since your last visit? |  |  |
| Have you been urinating more frequently since your last visit? |  |  |
| *Male ≥10 years*  Have you had ejaculations since the last visit? |  |  |
| *Female ≥10 years* Have you had your period since your last visit? |  |  |
| *Aged ≥14 years*  Are you sexually active? |  |  |
| *Aged ≥14 years*  Do you hope to have children in the future? |  |  |
| **Intoxications** |  |  |
| *Aged ≥14 years* |  |  |
| Do you drink alcohol? If yes, how often? |  |  |
| Do you smoke? |  |  |
| Do you use drugs? |  |  |
| Do you use any other substances that are important to mention? |  |  |
| **Psychosocial** |  |  |
| Do you need extra support at school or work? |  |  |
| How is your living situation? | *open response* | |

Supplementary Table 3. Topic guide participants

| - Introduction of the study   - Explanation of the study   - Estimated duration of the interview: 20-30 minutes   - Recording of the interview and privacy (informed consent) - Introduction of the patient   - In (year of HSCT) you have had a HSCT because of (diagnosis). Today/last week you have had a consultation with doctor (name clinician). - What did you think of your last consultation at the outpatient clinic? - Did you prepare yourself for the consultation? If so, how did you prepare? - Before the consultation you have been asked to complete online questionnaires about your well-being. Did you complete the questionnaires? If so:   - What did you think of the questionnaires?   - Did you find the questionnaires useful?   - In what setting did you complete the questionnaires (at home or somewhere else, alone or with help)? - If the parents are present: There were questionnaire for the parents as well, did you complete these questionnaires? If so:   - What did you think of these questionnaires?   - Did you find the questionnaires useful?   - In what setting did you complete the questionnaires (at home or somewhere else, alone or with help)?   - Have you discussed the questionnaires with the doctor? If so:     - How did you discuss your given answers?     - How did you feel about this?     - Did you understand everything that has been discussed?     - Would you have wanted to discuss it differently?   - If not, how did you feel about this? - You visit the outpatient clinic annually. If you compare this consultation with your previous consultation, how do you feel about this consultation? - Are you able to discuss everything that you want during your consultation with your healthcare provider?   - If not, what would help you to discuss everything with your healthcare provider? - How can we improve the consultations at the outpatient clinic?   [Additional questions]   - How do you feel when you have a consultation at the outpatient clinic?   - How does this affect you?   - Do you look forward to the consultation or not?   - Was it better than expected or worse? |
| --- |

Supplementary Table 4. Topic guide health care professionals

| - Introduction of the study   - Explanation of the study   - Recording of the interview and privacy (informed consent) - How do you feel about the consultations at the outpatient clinic in the last 3 months? - Could you describe the setting of a consultation from a patients’ perspective? - How do you prepare your consultations? - Patients are asked to complete online questionnaires in advance of the consultation. Do the patients complete the questionnaires? - What do you think about these questionnaires? - How do these questionnaires influence your consultation?   - Or: do you notice any difference in your consultation after PROM implementation? - Do you think the questionnaires are useful? - How do you discuss the questionnaires?   - How do you decide what to discuss?     - Or: do you adjust the way you discuss the questionnaires for each individual patient?   - How does the patient respond to this?     - Or: does this influence your relationship with the patient? If so, how do the questionnaires affect this?   - Do you feel barriers to discuss everything freely from the questionnaires? - When seeing pediatric patients:   - How do you discuss the questionnaires if the child and parents are both present at the consultation? - What do the questionnaires contribute to the consultation? - If the patient didn’t complete the questionnaire, how does this affect your consultation? - Are there any other topics that we have not discussed yet? |
| --- |

*Supplementary Table 5. Qualitative Analysis Guide of Leuven (1)*

| **Stage** | **Process** |
| --- | --- |
| **1** | Thorough (re)reading of the interviews |
| **2** | Narrative interview report |
| **3** | From narrative report to conceptual interview scheme |
| **4** | Fitting-test of the conceptual interview schemes |
| **5** | Constant comparison process |
| **6** | Draw up a list of concepts |
| **7** | Coding process – back to the ‘ground’ |
| **8** | Analysis and description of concepts |
| **9** | Extraction of the essential structure |
| **10** | Description of the results |

Supplementary Table 6. Details from non-participants (N=9)

| **Reason for not participating** | **N=9** |
| --- | --- |
| Appointment was postponed until after the inclusion period | 3 |
| Caregiver who completed the PROMs was not present at the consultation | 2 |
| Refused without providing a reason | 2 |
| Interview was cancelled due to unforeseen circumstances | 1 |
| PROs were not discussed due to technical problems | 1 |

Supplementary Table 7. Illustrative quotations from theme 5 ‘Usefulness of PROs’

| **Sex** | **Age** | **Quotation** |
| --- | --- | --- |
| *♂* | 17 | *[Caregiver] “Anyway, we did explain that these questions are asked now intentionally, because there are plenty of children after a transplantation that do, uhm, for who those questions are indeed relevant.”* |
| *♀* | 30 | *“And then there are questions about transplantation related stuff that would have happened recently, but well, for me it’s already 27 years ago, so for me it’s not recent anymore.”* |
| *♂* | 11 | *[Caregiver about completing the PROs] “I always like to participate in research, then I’ll think, we’ll help the next generation HSCT patients with this and hope that it will get better every time in doing so.”* |
| *♂* | 8 | *[Caregiver about the use of PROs] “I think it’s useful, because, well, for us it is not clear what causes certain things and it’s obviously also good to monitor the general wellbeing of a patient.”* |
| *♂* | 13 | *[Caregiver about the PROs regarding parents] “Well yeah, you know, it’s just going all right, fine and if it might be going a little bit less okay, I would also report that it’s going fine.”* |

Supplementary Table 8. Illustrative quotations from theme 6 ‘Opportunities for improvement of use of PROs’

| **Sex** | **Age** | **Quotation** |
| --- | --- | --- |
| *♀* | 16 | *“Sometimes there is a bit of uncertainty when asking about complaints in the last 7 days, why necessarily 7 days, like, I could have also had it another time.”* |
| *♂* | 17 | *[Caregiver] “You know what it is, the questionnaires are just a lot, this was a lot at once, especially when you are a teenager.“* |
| *♂* | 37 | *[About missing a positive approach in the PROs] “Well I get that most of the time doctors focus on what hurts and what isn’t going well, to help fix that, but sometimes it’s nice for a patient to see on what front they are doing better.”* |

Supplementary Table 9. Consolidated criteria for reporting qualitative studies (COREQ): 32-item checklist (2)

| ***No - Item*** | ***Guide question*** | ***Description*** |
| --- | --- | --- |
| **Domain 1: Research team and reflexivity** | | |
| **Personal Characteristics** | | |
| 1. Interviewer/facilitator | Which authors conducted the interview or focus group? | Methods, Measures: FZ and NG |
| 2. Credentials | What were the researcher’s credentials? | Title Page & Methods, Measures:  FZ: BSc student  NG: BSc, master student  JB: MSc, PhD student  HM: MSc, PhD student  AhP: PhD, cognitive psychologist |
| 3. Occupation | What was their occupation at the time of the study? | FZ: Bachelor student Educational Sciences  NG: Master student Medicine  JB: PhD student Pediatrics  HM: Pediatric hematology nurse and PhD-student  AP: Associate professor doctor-patient decisions |
| 4. Gender | Was the researcher male or female? | Female. |
| 5. Experience and training | What experience or training did the researcher have? | Title Page & Methods, Measures: BSc and MSc. |
| **Relationship with participants** | | |
| 6. Relationship established | Was a relationship established prior to study commencement? | Methods, Measures: no. |
| 7. Participant knowledge of the interviewer | What did the participants know about the researcher? | Methods, Measures:  independent researcher. |
| 8. Interviewer characteristics | What characteristics were reported about the interviewer/facilitator? | Methods, Measures: independent researcher, who did not have any (treatment) relationship with the participants. |
| **Domain 2: study design** | | |
| **Theoretical framework** | | |
| 9. Methodological orientation and Theory | What methodological orientation was stated to underpin the study? | Methods, Design: multiple methods study.  Methods, Analysis: thematic analyses. |
| **Participant selection** | | |
| 10. Sampling | How were participants selected? | Measures, Participants: all patients that met the inclusion criteria were approached. |
| 11. Method of approach | How were participants approached? | Measures, Participants: participants were approached by telephone [JB] and were given complete study information. |
| 12. Sample size | How many participants were in the study? | Results: 24 patients were approached of which 16 patients agreed to participate. One participant was excluded due to technical issues with completing the PROMs. |
| 13. Non-participation | How many people refused to participate or dropped out? Reasons? | Results: Of the 24 patients, three participants had postponed appointments outside inclusion period; two did not provide a reason; two could not participate, because the caregiver who filled in the questionnaire was not present at the consultation; one interview was cancelled due to illness of an interviewer; and one participant was excluded because the questionnaires were not discussed due to technical problems. Making it a total of 15 participants. |
| **Setting** | | |
| 14. Setting of data collection | Where was the data collected? | Methods, Measures: within the consultation room or by videoconference. |
| 15. Presence of non-participants | Was anyone else present besides the participants and researchers? | Results: seven interviews were held with only the participant of which four adults and three minors, four with both participant and his or her caregiver and four with only the caregiver of the participant. |
| 16. Description of sample | What are the important characteristics of the sample? | Results: The 15 participants had a median age of 17 years and the median age at HSCT was 3 years. Eight out of 15 participants were male (table 1). |
| **Data collection** | | |
| 17. Interview guide | Were questions, prompts, guides provided by the authors? Was it pilot tested? | Methods, Measures: semi-structured interviews by using a topic guide (Supplementary table 2). |
| 18. Repeat interviews | Were repeat interviews carried out? If yes, how many? | None |
| 19. Audio/visual recording | Did the research use audio or visual recording to collect the data? | Methods, Analysis: all interviews were recorded and transcribed verbatim. |
| 20. Field notes | Were field notes made during and/or after the interview or focus group? | No. |
| 21. Duration | What was the duration of the interviews or focus group? | Results: median interview duration was 21 minutes (range 11-46). |
| 22. Data saturation | Was data saturation discussed? | Methods, Analysis: Data collection continued until data saturation was reached, which was defined as no new upcoming themes in the analysis of three consecutive interviews. Data saturation was reached after 15 interviews. |
| 23. Transcripts returned | Were transcripts returned to participants for comment and/or correction? | No. |
| **Domain 3: analysis and findings** | | |
| **Data analysis** | | |
| 24. Number of data coders | How many data coders coded the data? | Methods, Analysis: three researchers (JB, FZ, NG) |
| 25. Description of the coding tree | Did authors provide a description of the coding tree? | No. |
| 26. Derivation of themes | Were themes identified in advance or derived from the data? | Methods, Analysis: derived from the data. |
| 27. Software | What software, if applicable, was used to manage the data? | Methods, Analysis: ATLAS.ti |
| 28. Participant checking | Did participants provide feedback on the findings? | No. |
| **Reporting** | | |
| 29. Quotations presented | Were participant quotations presented to illustrate the themes / findings? Was each quotation identified? | Results: Table 2-5, Supplementary Tables 6-7, illustrative quotes. |
| 30. Data and findings consistent | Was there consistency between the data presented and the findings? | Results: Table 2-5, Supplementary Tables 6-7, illustrative quotes. |
| 31. Clarity of major themes | Were major themes clearly presented in the findings? | Results: PROs 1) help to discuss topics; 2) make the patients feel understood; 3) create a moment of self-reflection; 4) make consultations more efficient |
| 32. Clarity of minor themes | Is there a description of diverse cases or discussion of minor themes? | Results: Table 2-5, Supplementary Tables 6-7, illustrative quotes. |

**References**

1. Dierckx de Casterlé B, Gastmans C, Bryon E, Denier Y. QUAGOL: a guide for qualitative data analysis. Int J Nurs Stud. 2012;49(3):360-71.

2. Tong A, Sainsbury P, Craig J. Consolidated criteria for reporting qualitative research (COREQ): a 32-item checklist for interviews and focus groups. Int J Qual Health Care. 2007;19(6):349-57.
